# Heterogeneity in health funding and disparities in health outcome: a comparison between high focus and non-high focus states in India

**DOI:** 10.1186/s12962-023-00451-x

**Published:** 2023-07-17

**Authors:** Ranjan Kumar Mohanty, Deepak Kumar Behera

**Affiliations:** 1 Xavier Institute of Management, XIM University, Bhubaneswar, Odisha 751013 India; 2 Department of Economics and Finance, The Business School, RMIT University, 700000 Ho Chi Minh City, Vietnam

**Keywords:** Publicly financed health expenditure, Health outcomes, Infrastructural development, High-focus states, Non-high-focus states, Fixed effects model, Random effects model, H51, I10, I18, C23

## Abstract

**Background:**

The Central Government of India introduced the National Health Mission (NHM) in 2005 to improve health outcomes by enhancing publicly financed (government) health expenditure and health infrastructure at the state level. This study aims to examine the effects of the state-level heterogeneity in publicly financed spending on health services on major health outcomes such as life expectancy, infant mortality rate, child mortality rate, the incidence of malaria, and immunization coverage (i.e., BCG, Polio, Measles, and Tetanus).

**Methods:**

This study investigates the relationships between publicly financed health expenditure and health outcomes by controlling income and infrastructure levels across 28 Indian States from 2005 to 2016. Along with all states, the empirical analysis has also been carried out for high-focus and non-high-focus states as per the NHM fund flow criteria. It has applied panel fixed-effects and random effects model wherever required based on the Hausman test.

**Results:**

The empirical results show that publicly financed health expenditure reduces infant mortality, child mortality, and malaria cases. At the same time, it improves life expectancy and immunization coverage in India. It also finds that the relationship between publicly financed health expenditure and health outcomes is weak, especially in the high-focus states.

**Conclusions:**

Given the healthcare need for achieving desirable health outcomes, Indian States should enhance publicly financed expenditure on health services. This study augments essential guidance for implementing public health policies in developing countries.

**Supplementary Information:**

The online version contains supplementary material available at 10.1186/s12962-023-00451-x.

## Background

With the transition from Millennium Development Goals (MDGs) to Sustainable Development Goals (SDGs), the literature on publicly financed health expenditure (PHE) and health outcomes has attracted the attention of researchers and policymakers around the globe especially in developing countries. Studies on the linkage between PHE and health outcomes guide the implementation of public health policies in developing countries. India, an emerging and developing economy, has taken several initiatives to augment PHE since 2000. It adopted the MDGs^1^ in September 2000, setting various health targets like reducing infant and child mortality, improving maternal health, combating HIV/AIDS, Malaria cases, and other deadly diseases. It introduced the ‘National Rural Health Mission (NRHM)’ in 2005 to improve various proximate and ultimate health outcomes with an increase in PHE.^2^ Similarly, an insurance scheme named ‘Rashtriya Swasthya Bima Yojana (RSBY)’ was introduced in 2008 across Indian states. A high-level expert group on Universal Health Coverage (UHC) in 2011 recommended an increase in PHE to 2.5 per cent of Gross Domestic Product (GDP) by 2017 and 3 per cent of GDP by 2022 in India.^3^ National Urban Health Mission (NUHM) was launched in 2013.^4^ The Ayushman Bharat Mission was launched in 2018.^5^ These back-to-back measures implemented by Indian Governments in recent times are intended to improve the performance of the hitherto neglected health sector.

**Fig. 1:**
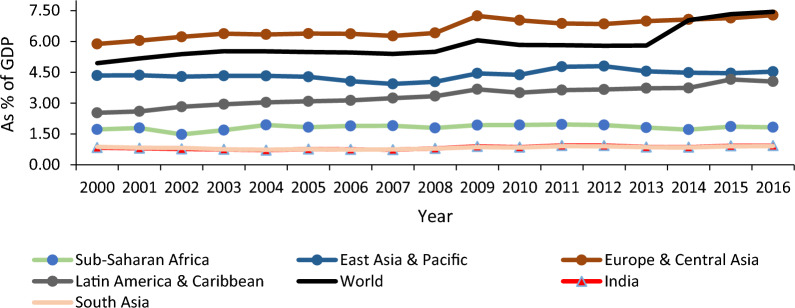
Publicly financed health expenditure as percent of GDP. *Source*: World Development Indicators, World Bank

**Fig. 2:**
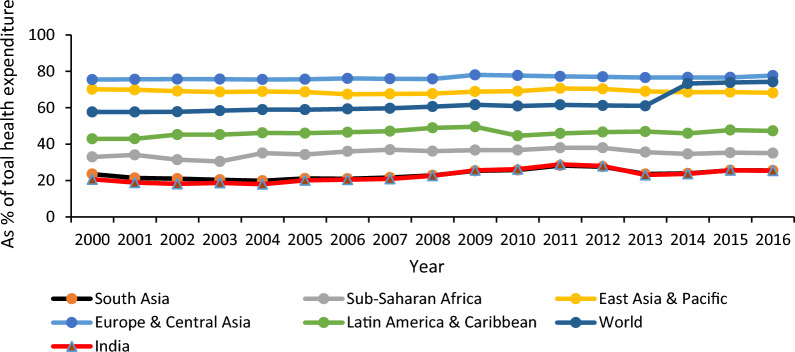
Publicly financed health expenditure (% of total health expenditure). *Source*: World Development Indicators, World Bank

Despite an increased PHE in recent times, India has partially achieved the target set by the MDGs.^6^ India’s position concerning health indicators is abysmal (its rank lies in the bottom 30 per cent group) as per the Human Development Report-2018. It is believed that the low level of PHE might be one of the possible causes for India’s relatively worse performance on health indicators [1, 2]. India has been experiencing a meager share of PHE in its GDP and total health expenditure (Figs. 1, 2). The low level of PHE has mainly resulted in poor health infrastructure in India [3]. Other regions like Europe & Central Asia, East Asia & Pacific, Latin America & Caribbean, etc., and even Sub-Saharan Africa have a much higher share of PHE in their GDP and total health expenditure than India. Specifically, India’s share of PHE in GDP has remained almost stagnant (hovering around 1 per cent) from 2000 to 2016. PHE constituted slightly more than one-fourth of its total health expenditure during this period. PHE has been almost three-fourths of their total health expenditure in other regions like Europe, Central Asia, and East Asia & Pacific. Even Sub-Saharan Africa has more than one-third share of PHE.

The impact of PHE on improving/deteriorating health status has received relatively less attention in the literature. Government intervention in the healthcare sector is necessary. It has been argued on several grounds, such as positive externalities associated with health and the inability of private markets to meet existing demand for healthcare [4]. The literature on the linkage between publicly financed expenditure on health and health outcome have mixed opinions: some studies find a positive effect [3, 5–7], while other studies find a negative or statistically insignificant effect on health outcomes [8–12]. PHE may improve health outcomes via higher access to health care or worsen the health outcome if it leads to inefficient production of health care services [13]. Similarly, an increase in out-of-pocket health expenditure could have an enormous adverse impact on patients, particularly those with low incomes, leading to low utilization of healthcare services and later resulting in poor health status [14].

From the policy perspective, if an increase in PHE positively impacts health outcomes, India could rapidly achieve a better health status by enhancing its PHE. But there is a persistent gap between health-related developmental outcomes in the Indian states due to limited fiscal space, low spending priority, less absorption capacity, and spending inefficiency [15–17]. The crucial question is whether PHE has any significant effects on health outcomes in Indian states. Does it affect the health outcomes adversely or favorably? Which factors are essential for improving health outcomes in India? Does the effect of PHE on health outcomes vary across different categories of states? The current study is motivated by the inconclusive debate on the relationship between PHE and health outcomes, particularly in the Indian states.

The literature on this crucial issue among the Indian states is scarce. Under the Indian Constitutional structure, state Governments have predominant responsibility for providing health care services in India.^7^ The central government's role is to assist or supplement the health expenditure of the states.^8^ Based on resource availability, priority, fiscal space, etc., there is a massive variation in per capita publicly financed health expenditure (PCPHE) and the health indicators across states. Additionally, the availability of data across states is consistent and comparable. Investigating the health outcomes in the Indian states is very crucial for achieving SDGs targets and moving towards the UHC. This study will help the policymakers to implement public health policies as these classifications are based on the states' prevalence of health outcome indicators.

To the best of our knowledge, this is one of the earliest studies to verify the effects of PHE on health outcomes across 28 Indian states from 2005 to 2016.^9^ Most studies have focused on the impact of PHE on a single indicator, i.e., Infant Mortality Rate (IMR). None of the studies has examined this issue by using diverse health outcomes like life expectancy at birth, IMR, Child Mortality Rate (CMR), and other preventive and curative diseases such as Incidence of Malaria, various immunization coverage (i.e., BCG, Polio, Tetanus, and Measles). This study fills this gap by examining the effect of PHE on diverse health outcomes across Indian states. However, along with PHE, other factors like income level, health infrastructure, demographic characteristics, state-specific factors, etc., have a more significant impact on improving health outcomes, partly addressed (based on data availability) in this study. Along with all states, this issue is also examined by dividing Indian states into the High-Focus States (HFS) and Non-High Focus States (NHFS) to verify the differential impact of PHE on health outcomes, which is a novel attempt.

Following the introduction, the remaining part of the study is structured as follows. The data and analytical framework of the study has reported in methodology section. Trends analysis and empirical estimation are presented in the results section. Critical analysis of empirical results has reported in the discussion section. Final section contains conclusion and policy implications.

## Methods

### Data

This study has used annual panel data for 28 Indian states for the period from 2005 to 2016.^10^ It will enable us to capture the effectiveness of an increase in PHE due to the launch of the National Health Mission (NHM)^11^ and the enactment of the MDGs in the Indian health system. The starting period of the data (i.e., 2005) is chosen based on introducing the NHM and the MDGs in India. The empirical analysis is extended to both HFS & NHFS to assess the differential impact of PHE on health outcomes.^12^ The variables considered are PHE,^13^ Gross State Domestic Product (GSDP), population, health service infrastructure,^14^ infant mortality rate (IMR),^15^ child mortality rate (CMR),^16^ life expectancy at birth (LE), incidence of malaria,^17^ and immunisation achieved.^18^

The data on PHE is collected from the National Institute of Public Finance and Policy (NIPFP) databank and the ‘State Finances: A study of budget’ by the Reserve Bank of India (RBI). Data on GSDP is obtained from National Accounts Statistics, Central Statistics Office (CSO). Data on population, health service infrastructure, malaria cases, and various immunizations achieved are from the EPW Research Foundation (EPWRF) India Time Series, and the data on health-related indicators such as IMR, CMR, and LE are collected from the Sample Registration System (SRS) Bulletins, Office of the Registrar General & Census Commissioner (ORGCC), Government of India (GOI). The variable description is presented in the Additional file 1: Table S1.

The summary statistics, and pair-wise correlation of selected variables are presented in Table 1. It shows that PCPHE and per capita GSDP (PCGSDP) are positively correlated with life expectancy and negatively correlated with IMR, CMR, immunization, and malaria in the Indian states. Health infrastructure has a positive relationship with immunization and malaria. Since correlation coefficients are not very informative, we apply the econometric technique for measuring genuine relationships.

**Table 1:** Descriptive statistics, and pair-wise correlations of variables of Indian states. *Source*: Author’s calculation

Variables	Descriptive statistics			Correlation		
	Obs.	Mean	SD	PCPHE	PCGSDP	INFRA
*All states*						
LE	204	67.5	3.5	0.62	0.66	0.11
IMR	336	37.8	16.0	− 0.53	− 0.56	− 0.11
CMR	228	11.9	5.2	− 0.52	− 0.68	− 0.01
MALARIA	336	2.8	4.7	0.20	− 0.11	0.41
IMMU	336	18.52	4.7	− 0.49	− 0.43	− 0.15
BCG	336	19.56	4.8	− 0.47	− 0.48	− 0.16
MEASLES	336	18.10	4.4	− 0.47	− 0.41	− 0.15
POLIO	336	18.34	4.6	− 0.45	− 0.40	− 0.12
TETANUS	336	18.08	5.7	− 0.50	− 0.39	− 0.14
PCPHE	336	584.3	474.4	…	…	…
PCGSDP	336	47,030.8	28,898.4	…	…	…
INFRA	336	0.21	0.1	…	…	…
*High focus states*						
LE	96	65.7	3.6	0.81	0.67	0.53
IMR	216	41.4	16.1	− 0.63	− 0.51	− 0.41
CMR	120	14.9	4.4	− 0.71	− 0.66	− 0.42
MALARIA	216	9.1	2.6	0.15	0.01	0.36
IMMU	216	18.7	4.8	− 0.55	− 0.47	− 0.44
BCG	216	20.0	5.0	− 0.54	− 0.54	− 0.46
MEASLES	216	18.3	4.5	− 0.55	− 0.48	− 0.46
POLIO	216	18.5	4.7	− 0.51	− 0.44	− 0.40
TETANUS	216	19.9	5.7	− 0.51	− 0.37	− 0.39
PCPHE	216	655.3	517.0	…	…	…
PCGSDP	216	38,265.4	18,557.7	…	…	…
INFRA	216	7.7	1.4	…	…	…
*Non-high focus states*						
LE	108	69.2	2.5	0.62	0.43	− 0.29
IMR	120	31.4	13.7	− 0.66	− 0.58	0.27
CMR	108	8.6	3.7	− 0.37	0.19	0.98
MALARIA	120	9.6	1.5	0.15	0.14	− 0.14
IMMU	120	18.1	4.6	− 0.43	− 0.49	0.66
BCG	120	18.7	4.5	− 0.43	− 0.48	0.59
MEASLES	120	17.6	4.3	− 0.37	− 0.43	0.66
POLIO	120	17.9	4.4	− 0.38	− 0.44	0.68
TETANUS	120	18.3	5.6	− 0.51	− 0.57	0.67
PCPHE	120	456.5	353.5	…	…	…
PCGSDP	120	62,808.4	36,582.2	…	…	…
INFRA	120	8.6	1.2	…	…	…

Definition and descriptive of variables are reported in the supplementary file (Additional file 1: Table S1); SD: Standard Deviation

### Analytical framework

The primary objective of this study is to examine the impact of PHE on health outcomes in the Indian States. Thus, the variable of our interest is PHE. It assumes that higher PHE would lead to a higher life expectancy and lower IMR and CMR. The study has included other control variables that might impact health outcomes along with PHE.^19^ Other selected variables are PCGSDP and total health service infrastructure, following the literature [11, 18]. It is believed that higher per capita income is expected to have a favorable impact on health outcomes as states can prioritize their health expenditure due to enhanced fiscal capacity. It is likely that wealthier individuals, on average, can invest/spend more on medical expenses, prefer a healthier diet, lead a healthier lifestyle, and have lower morbidity rates than individuals with less income. Thus, higher per capita income is expected to have a favorable impact on health outcomes. Availability of health infrastructure provides easy access and affordable health care facility, which helps improve people's health status. Thus, along with PHE, per capita income and health service infrastructure are also added as explanatory variables in the regression model as follows.^20^

The Model:

1 $$Y_{it} = \alpha_i + \beta_1 X_{it} + \beta_2 Z_{it} + v_i + \in_{it}$$

where ‘i’ represents states, and “t” refers to time. ${Y}_{it}$ represents all the selected health outcomes like life expectancy, infant mortality rate, child mortality rate, malaria cases, and immunization. ${X}_{it}$ is the variable of interest, i.e., PCPHE. ${Z}_{it}$ embodies the other selected explanatory variables like PCGSDP and health infrastructure (INFRA). $\alpha_i$ represents intercept or constant. ${\text{v}}_{\text{i}}$ shows the effects of excluded variables in the model that are invariant over time and might impact the state’s health outcome. It is assumed that the state-specific effects ${\text{v}}_{\text{i}}$ are fixed rather than random. In this study, some unobserved factors, such as changing technology and medical practices, literacy level, other health infrastructures, government policies, etc., could improve health outcomes. Therefore, the model is called an unobserved effects model or a fixed-effect model (FE).^21^${\upvarepsilon }_{{\text{it}}}$ is an error term, often called the idiosyncratic error or time-varying error because it represents unobserved factors that change over time and affect${Y}_{it}$.

The goal of using a fixed effect is to eliminate ${\text{v}}_{\text{i}}$ because we believe it correlates with one or more ${\text{x}}_{{\text{it}}}$. But suppose we find that ${a}_{i}$ is uncorrelated with or independent of any explanatory variables in all periods, the Eqs. (2–10) become a random-effects model (RE). Comparing the FE and RE estimates whether there is a correlation between the ${\text{v}}_{\text{i}}$ and the ${\text{x}}_{{\text{it}}}$, it also assumes that the idiosyncratic errors and explanatory variables are uncorrelated across all periods. Hence, it can be verified through the Hausman test. The Hausman test results suggest both fixed and random effect models based on different regression specifications. Further, we have done a series of unit-root tests to verify whether any time-series effects exist in the fixed effects model or not. Our unit-root test shows that variables are level stationary (see Additional file 1: Table S2), indicating an absence of time effects in the regression model.

The estimated panel regression Eqs. (2–10) are as follows^22^:

2 $$lnLE_{it} = \alpha_i + \beta_1 lnPCPHE_{it} + \beta_2 lnPCGSDP_{it} + \beta_3 lnINFRA_{it} + v_i + \in_{it}$$

3 $$lnIMR_{it} = \alpha_i + \beta_1 lnPCPHE_{it} + \beta_2 lnPCGSDP_{it} + \beta_3 lnINFRA_{it} + v_i + \in_{it}$$

4 $$\ln CMR_{it} = \alpha_i + \beta_1 lnPCPHE_{it} + \beta_2 lnPCGSDP_{it} + \beta_3 lnINFRA_{it} + v_i + \in_{it}$$

5 $$\ln MALARIA_{it} = \alpha_i + \beta_1 lnPCPHE_{it} + \beta_2 lnPCGSDP_{it} + \beta_3 lnINFRA_{it} + v_i + \in_{it}$$

6 $$\ln IMMU_{it} = \alpha_i + \beta_1 lnPCPHE_{it} + \beta_2 lnPCGSDP_{it} + \beta_3 lnINFRA_{it} + v_i + \in_{it}$$

7 $$\ln BCG_{it} = \alpha_i + \beta_1 lnPCPHE_{it} + \beta_2 lnPCGSDP_{it} + \beta_3 lnINFRA_{it} + v_i + \in_{it}$$

8 $$\ln POLIO_{it} = \alpha_i + \beta_1 lnPCPHE_{it} + \beta_2 lnPCGSDP_{it} + \beta_3 lnINFRA_{it} + v_i + \in_{it}$$

9 $$\ln MEASLES_{it} = \alpha_i + \beta_1 lnPCPHE_{it} + \beta_2 lnPCGSDP_{it} + \beta_3 lnINFRA_{it} + v_i + \in_{it}$$

10 $$\ln TETANUS_{it} = \alpha_i + \beta_1 lnPCPHE_{it} + \beta_2 lnPCGSDP_{it} + \beta_3 lnINFRA_{it} + v_i + \in_{it}$$

where $lnLE$: log of life expectancy at birth; $lnIMR$: log of infant mortality rate; $lnCMR$: log of child mortality rate; $lnMALARIA$: log of malaria cases per 1000 population; $lnIMMU$: log of per capita immunization rate; $lnPCPHE$: log of per capita publicly financed expenditure on health services; $lnINFRA$: log of total health infrastructure per 1000 population; $\ln BCG$: log of per capita BCG immunisation for children achieved rate; $\ln POLIO$: log of per capita polio immunisation achieved rate; $\ln MEASLES$: log of per capita measles immunisation achieved rate; $\ln TETANUS$: log of per capita tetanus immunisation achieved rate. Definitions of variables are presented in Additional file 1: Table S1. Therefore, this study has divided the health outcomes into two categories: ultimate and proximate outcomes. The life expectancy, IMR, and CMR are considered under ultimate health outcomes, while variables like cases of malaria, and immunizations coverage are considered under proximate targets.

## Results

### Trends in per capita publicly financed health expenditure and health outcomes in India

**Fig. 3:**
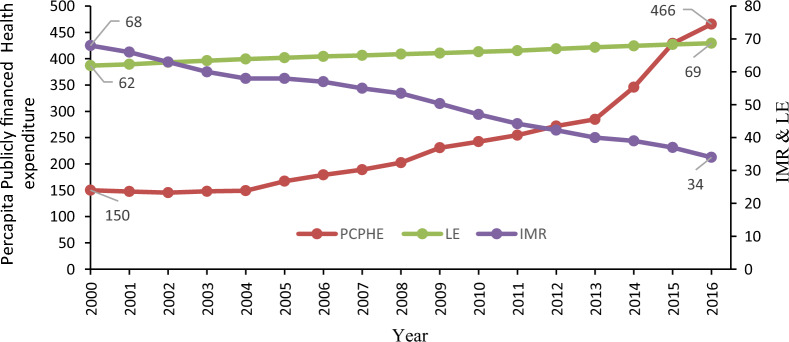
Trends in per capita publicly financed health expenditure, life expectancy, and infant mortality rate in India. *Note*: PCPHE-Per capita publicly financed expenditure on health, LE-Life expectancy at birth, and IMR-Infant Mortality rate. *Source*: Author’s calculations

**Fig. 4:**
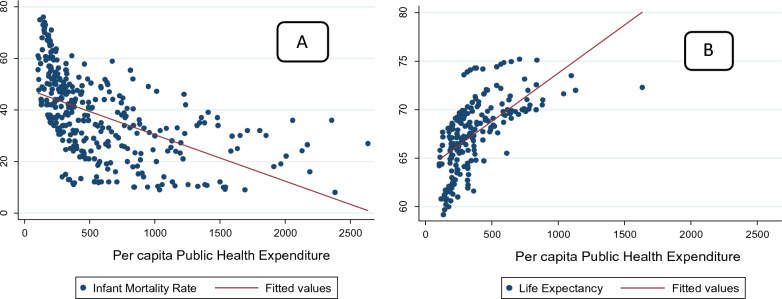
Scatter plots of (**A**) per capita publicly financed health expenditure, infant mortality rate, and (**B**) life expectancy across the Indian states using data for 2005–2016. *Source*: Author's calculations

The trends in PCPHE, IMR, and LE in India are shown in Figs. 3 and 4. It indicates that PCPHE was stagnant up to 2004 and then started increasing, which might be due to the introduction of NRHM in the Indian economy in 2005. The PCPHE has raised more than threefold during 2000–2016. LE has also increased from nearly 62 to 69 years during this period. IMR has shown a downward trend from 68 to 34 during this time. Thus, a preliminary observation shows a positive relationship between PCPHE and LE and a negative relationship between PCPHE and IMR in India. The inter-state inequalities in health expenditure have increased as there is a wide variation in the PCPHE across the Indian states [1, 16]. The scatterplots of PCPHE against IMR and LE for all the selected states from 2000 to 2016 are shown in Fig. 4. A linear regression line is also included in the graph. Figure 4 shows that states with higher PCPHE have witnessed lower IMR (the line slopes downward in “A”), while higher PCPHE states have seen higher LE (the line slopes upward in “B”).

### Unit-root test results

Before regression estimation, checking for stationarity is essential. Therefore, we have estimated the stationarity properties of the selected variables using Levin, Lin & Chu [19], and Im-Pesaran-Sin [20] panel unit root tests. The results of LLC and IPS unit root tests have been reported in Additional file 1: Table S2. The unit root test results show that variables do not reject the null hypothesis of no unit root, indicating that the selected series are stationary at level. So, we can apply a short-run regression model with no time-series effects to examine the dependence of one variable (i.e., dependent) on other or more variables (i.e., independent).

### Regression results: all states

Table 2 presents the regression results between PHE and selected health outcomes (i.e., LE, IMR, CMR, MALARIA, and IMMU)^23^ by controlling PCGSDP and INFRA in selected 28 states of India. Then, a similar kind of analysis has also been done separately for the HFS and NHFS of India (see Tables 3 and 4). Further, we have estimated separate models for individual vaccine coverage for immunization (see Eq. 7–10).^24^

The results of all the states (Table 2) show that PCPHE has a positive and significant impact on LE and IMMU. It implies that a one per cent increase in PCPHE leads to an increase of 0.021 per cent in the LE and a 0.19 per cent increase in the IMMU in the Indian states. However, an increase in PCPHE has an adverse impact on IMR, CMR, and Malaria incidence cases. It implies that a one per cent increase in PCPHE leads to a 0.15, 0.08, and 0.62 per cent reduction in the IMR, CMR, and MALARIA, respectively. Our results are similar to those of earlier studies that have argued that PHE positively impacts life expectancy. It reduces child mortality, increases immunization coverage, and prevents deadly diseases [21–24].

The results also show that PCGSDP has a positive and statistically significant impact on LE. It has an adverse effect on IMR, CMR, and MALARIA. It implies that an increase in the state’s income could mobilize PHE, nutrition, better sanitation, etc., eventually improving health indicators. We have found that the availability of health infrastructure reduced child mortality by around 0.5 percent. The infrastructure has no significant impact on other health outcome parameters. On the contrary, some studies found that only PHE could not achieve the potential health outcomes; it requires the support of health services infrastructure and per capita income [25–27]. Along the same lines, we have also found that health services infrastructure plays a crucial role in reducing the under-5 mortality rate in Indian states.

**Table 2:** Results of panel multiple regression using fixed and random effects model in the sample of Indian states. *Source*: Author’s calculation

Variables	lnLE_it_	lnIMR_it_	lnCMR_it_	lnMALARIA_it_	lnIMMU_it_	lnBCGI_it_	lnMEASLES_it_	lnPOLIO_it_	lnTETANUSit
lnPCPHE_it_	0.021***	− 0.153***	− 0.076*	− 0.618***	0.115***	0.100***	0.098**	0.098**	0.160***
	(0.004)	(0.041)	(0.040)	(0.158)	(0.037)	(0.038)	(0.038)	(0.042)	(0.050)
lnPCGSDP_it_	0.061***	− 0.421***	− 0.890***	− 0.533**	− 0.486***	− 0.517***	− 0.406***	− 0.459***	− 0.539***
	(0.007)	(0.060)	(0.071)	(0.234)	(0.055)	(0.057)	(0.057)	(0.063)	(0.075)
lnINFRA_it_	− 0.013	− 0.050	− 0.491***	0.454	0.077	0.040	0.146*	0.106	− 0.033
	(0.010)	(0.086)	(0.102)	(0.326)	(0.079)	(0.081)	(0.081)	(0.090)	(0.106)
Constant	3.431***	8.844***	11.284***	9.703***	7.479***	7.886***	6.826***	7.334***	7.536***
	(0.052)	(0.439)	(0.488)	(1.742)	(0.403)	(0.415)	(0.411)	(0.459)	(0.544)
Hausman test	1.55	19.18***	8.97**	2.70	20.42***	14.95***	18.01***	17.50***	23.51***
State’s FE	NO	Yes	Yes	No	Yes	Yes	Yes	Yes	Yes
R-squared	0.837	0.596	0.848	0.336	0.150	0.182	0.100	0.110	0.101
F-test	966.02***	149.69***	384.65***	153.00***	58.34***	68.81***	41.22***	43.82***	31.05***
No. of obs	204	336	228	336	336	336	336	336	336
No. of States	17	28	19	28	28	28	28	28	28

ln: Natural logarithm; Standard errors in parentheses, ***p < 0.01, **p < 0.05, *p < 0.1. Significant probability (*p*) values in the Hausman test represent the application of fixed effects (FE) models, while other regression specifications use random effects (RE) models. Country FE: NO—RE; Yes—FE

### Regression results: high focus states (HFS)

Similarly, the results for the HFS (Table 3) find that PCPHE has a positive and statistically significant impact on LE and IMMU. It implies that a one per cent increase in PCPHE leads to an increase of LE by 0.03 per cent and a slightly higher than a one per cent increase in the IMMU. It also finds that an increase in PCPHE helps in reducing the IMR and MALARIA among HFS. A one per cent increase in PCPHE leads to 0.11 and 0.59 per cent reduction in the IMR and MALARIA, respectively. It finds an insignificant effect of PCPHE on the under-5 mortality rate in HFS.

**Table 3:** Results of panel multiple regression using fixed and random effects model in the sample of high focus states in India. *Source*: Author’s calculations

Variables	lnLE_it_	lnIMR_it_	lnCMR_it_	lnMALARIA_it_	lnIMMU_it_	lnBCGI_it_	lnMEASLES_it_	lnPOLIO_it_	lnTETANUSit
lnPCPHE_it_	0.034***	− 0.107**	− 0.063	− 0.586***	0.079*	0.051	0.070	0.067	0.123**
	(0.005)	(0.049)	(0.046)	(0.180)	(0.046)	(0.048)	(0.047)	(0.053)	(0.062)
lnPCGSDP_it_	0.062***	− 0.406***	− 0.860***	− 0.184	− 0.414***	− 0.441***	− 0.366***	− 0.409***	− 0.394***
	(0.009)	(0.072)	(0.080)	(0.268)	(0.068)	(0.071)	(0.069)	(0.078)	(0.093)
lnINFRA_it_	− 0.011	− 0.210*	− 0.517***	1.111***	− 0.203*	− 0.213*	− 0.150	− 0.221*	− 0.274*
	(0.014)	(0.114)	(0.150)	(0.408)	(0.107)	(0.112)	(0.109)	(0.124)	(0.146)
Constant	3.339***	8.201***	10.942***	7.043***	6.411***	6.924***	6.031***	6.396***	5.753***
	(0.067)	(0.526)	(0.597)	(2.037)	(0.497)	(0.517)	(0.506)	(0.572)	(0.676)
Hausman test	1.48	16.47***	18.55***	0.23	9.28**	6.13*	8.17**	9.75**	11.11**
State’s FE	NO	Yes	Yes	NO	Yes	Yes	Yes	Yes	Yes
R-squared	0.890	0.507	0.844	0.171	0.010	0.112	0.100	0.077	0.071
F-test	697.58***	63.69***	184.51***	65.33***	24.70***	31.80***	18.64***	19.54***	8.99***
No. of obs	96	216	120	216	216	216	216	216	216
No. of States	8	18	10	18	18	18	18	18	18

ln: Natural logarithm; Standard errors in parentheses, ***p < 0.01, **p < 0.05, *p < 0.1. Significant probability (*p*) values in the Hausman test represent the application of fixed effects (FE) models, while other regression specifications use random effects (RE) models. Country FE: NO—RE; Yes—FE

As discussed earlier, PCGSDP has a favorable effect on LE and an adverse impact on IMR, CMR, and MALARIA in all states sample. But in HFS, we have found that malaria incidence has no relationship with PCGSDP. Similarly, higher availability of INFRA helps in reducing IMR (by 0.21 percent) and CMR (by 0.52 percent) in the HFS. Health infrastructure also helps in detecting the total number of MALARIA cases in these States. Our finding is similar to Fay et al. [28], who found that better access to basic infrastructure, which is complementary to health services infrastructure, has a large and statistically significant effect in reducing IMR, CMR, and stunting. The provision of health services infrastructure has no direct impact on the incidence of malaria and immunization in all the states. The infrastructure size is not the only way to achieve better health outcomes. The quality of health services remains a challenge in India's health system for advancing UHC [29].

### Regression results: non-high focus states (NHFS)

A similar empirical analysis is carried out for NHFS (Table 4). The empirical results show that PCPHE has a negative and significant impact on IMR, CMR, and MALARIA incidence while positively impacting IMMU coverage. It implies that a one percent rise in PCPHE leads to a fall in IMR by 0.336 percent. Additionally, it finds that a one percent increase in PCPHE leads to an increase in immunization coverage by 0.42 percent in a year. However, PCPHE does not have any significant effects on LE in NHFS.

**Table 4:** Results of panel multiple regression using fixed and random effects model in the sample of non-high focus states in India. *Source*: Author’s calculations

Variables	lnLE_it_	lnIMR_it_	lnCMR_it_	lnMALARIA_it_	lnIMMU_it_	lnBCGI_it_	lnMEASLES_it_	lnPOLIO_it_	lnTETANUSit
lnPCPHE_it_	− 0.004	− 0.336***	− 0.114	− 0.468	0.286***	0.324***	0.210***	0.229***	0.405***
	(0.007)	(0.065)	(0.094)	(0.313)	(0.045)	(0.047)	(0.051)	(0.050)	(0.056)
lnPCGSDP_it_	0.079***	− 0.324***	− 0.891***	− 1.609***	− 0.777***	− 0.852***	− 0.567***	− 0.658***	− 1.087***
	(0.012)	(0.097)	(0.166)	(0.465)	(0.067)	(0.070)	(0.076)	(0.075)	(0.083)
lnINFRA_it_	− 0.017	0.257**	− 0.492***	− 1.304***	0.593***	0.497***	0.710***	0.728***	0.371***
	(0.012)	(0.101)	(0.169)	(0.484)	(0.069)	(0.073)	(0.079)	(0.078)	(0.086)
Constant	3.371***	9.358***	11.421***	16.958***	10.790***	11.228***	9.144***	10.078***	13.045***
	(0.079)	(0.692)	(1.076)	(3.309)	(0.476)	(0.501)	(0.544)	(0.533)	(0.592)
Hausman test	18.54***	0.64	15.96***	5.57	24.97***	22.21***	19.43***	25.89**	29.37***
State’s FE	Yes	NO	Yes	NO	Yes	Yes	Yes	Yes	Yes
R-squared	0.593	0.829	0.873	0.593	0.706	0.694	0.593	0.666	0.692
F-test	235.19***	552.59***	204.52***	165.52***	158.52***	145.78***	90.61***	121.20***	149.31***
No. of obs	108	120	108	120	120	120	120	120	120
No. of States	9	10	9	10	10	10	10	10	10

ln: Natural logarithm; Standard errors in parentheses, ***p < 0.01, **p < 0.05, *p < 0.1. Significant probability (*p*) values in the Hausman test represent the application of fixed effects (FE) models, while other regression specifications use random effects (RE) models. Country FE: NO—RE; Yes—FE

On the other hand, PCGSDP positively impacts LE and negatively impacts IMR, CMR, and Malaria incidence. It implies that high PCGSDP reduces IMR, CMR, and MALARIA in NHFS. We have found that the higher availability of INFRA helps reduce CMR and MALARIA and increase the IMMU in NHFS, which is a very interesting insight in our study.

### Regression results: different types of vaccine coverage for immunization

In this study, we have estimated the effects of PHE on various immunizations by adopting four important vaccination coverage variables, including BCG, Measles, Polio, and Tetanus, using the fixed effects model. Tables 2, 3 and 4 (Column 6–10) presents the regression results of per capita immunization achieved rate across all states, HFS, and NHFS in India. It shows a positive association between PCPHE and vaccine coverage in all states, HFS and NHFS. There is a wide variation in coefficient values between HFS and NHFS in India. The elasticity of PCPHE with respect to vaccine coverage is less than one, and the coefficients vary from 0.079 percent to 0.123 percent if health expenditure increases at one percent per year in the HFS of India. On the other hand, the elasticity of PCPHE with respect to vaccine coverage is less than one, and the coefficient values vary from 0.210 percent to 0.405 percent if health expenditure increases by one percent per year in NHFS of India. Another interesting result is that increased PCGSDP reduces the government coverage of vaccination in all state categories. It could be due to income effects and people's health-seeking behavior, which declines government vaccine coverage.

Overall, we find that both PCPHE and PCGSDP have played significant roles in improving the selected health outcomes in the Indian States. But the elasticity of health outcomes (LE, IMR, and CMR) with respect to per capita income is much stronger than their elasticity concerning publicly financed expenditure on health services. However, in reducing malaria and increasing immunization coverage, PHE is more effective than per capita income.

## Discussions

The empirical results show that PHE has a positive and statistically significant effect on life expectancy and immunization. In contrast, it has a negative impact on infant mortality rate, child mortality rate, and malaria cases. Per capita income has an adverse effect on infant mortality rate, child mortality rate, and malaria cases, while it has a favorable impact on improving life expectancy across states. It also finds that total health services infrastructure can reduce the inequality in health outcomes among the states, irrespective of their levels of development. The study is very relevant on the way to achieve universal health coverage at the state level. PHE has a more significant impact on reducing infant and child mortality in high-focus states than in non-high-focus states. Life expectancy is significantly affected by PHE and state’s income in high focus states, while it is only influenced by state’s income among non-high focus states. Health infrastructure is more crucial in improving health outcomes in high-focus states than in non-high-focus states.

This phenomenal performance can be linked to enhancing the Central Government’s fund under the NHM since its inception in 2005–2006. Under the NHM, the Central Government contributes 60 per cent and 90 per cent of the total NHM funds among the General Category States (GCS) and Special Category States (SCS), respectively. Additional assistance by the Center helps the state Governments prioritize health care, which helps to improve the overall health outcomes such as a reduction in IMR and CMR, an increase in immunization, drop in malaria incidence due to fund flow in diseases specific programs. However, per capita income has a detrimental effect on the immunization rates across states, irrespective of their category. As income increases, people adopt birth control measures and prefer a maximum of one or two children. As the birth rate falls, immunization coverage declines among high-income people. It is also found that greater access to health care services leads to a reduction in child-health-related mortality.

Many cross-country studies find little effect of PHE on health outcomes. At the same time, income level plays a crucial role in determining better health status [30–32], and socio-economic factors are often highly associated with health outcomes [33]. India-specific studies on the impact of PHE on health outcomes are inconclusive [3, 6, 12, 24, 27, 34]. Barenberg et al. [24], using an unbalanced panel of 31 Indian states and Union Territories from 1983–1984 to 2011–2012, find that PHE helps in lowering the IMR among the Indian States. Farahani et al. [6] evaluate the relationship between state-level PHE and individual mortality across all age groups in India. They find that a 10% increase in PHE decreases mortality by about 2%, with effects mainly concentrated on women, the young, and the elderly. Deolalikar [12] found that PHE does not have a significant impact on mortality rates using the Indian states for 1980–1999, while Bhalotra [3] found a significant impact of PHE on IMR by using the sample of rural households. Some related studies examine the impact of decentralization on rural IMR in India [34] and the cyclicality of PHE in economic crises [27].

In the same argument, country-level experiences opine that healthcare transformation in public health funding policies improves patients' accessibility and financial protection in Iran [35]. Our study also finds an inequality in achieving health outcomes between HFS and NHFS states of India. Evidence from South African countries shows that positive effects of health funding on health outcomes could be better achieved through public–private partnerships (PPP) and cost-effectiveness of treatment of any diseases [36]. Few Indian studies argue that private health care expenditure is a major funding source for treating diseases, financially burdening people. Government-funded health insurance's effects are negligible in reducing out-of-pocket health expenditure [37]. They suggest the urgent need for political prioritization to design health system financing policies and provide financial risk protection for UHC [38]. Along similar lines, the literature argues that due to seeking quality care at private hospitals, people prefer to utilize private health care facilities for Tuberculosis treatment than publicly financed health facilities in India. Therefore, the health care burden on people has been increasing over time, which needs outcome-based funding in health care at publicly funded health facilities [39].

## Conclusions

The empirical linkage between publicly financed health expenditure and health outcomes is of interest to policymakers in India because of India's steady rise in per capita publicly funded health care expenditure. Thus, publicly financed health policies and direct intervention are required to prevent communicable and non-communicable diseases, namely, malaria and vaccination of children. At the same time, the ultimate health goals like life expectancy and child-health-related mortality are influenced by non-medical factors, particularly the standard of living and lifestyle. Per capita publicly financed health expenditure has a more significant impact on reducing infant and child mortality in HFS than in NHFS. Life expectancy is significantly affected by publicly funded health expenditure and income in HFS, while it is only influenced by income in NHFS. Health infrastructure is more crucial in improving health outcomes in HFS than in NHFS.

State governments of India have already started efforts to reduce mortality and achieve SDGs by 2030 in different health parameters such as preventing and treating malaria, providing safe drinking water, proper sanitation, nutrition, etc., through Swachha Bharat Mission. All these steps could help improve health status, reducing mortality among infants, children, and adults. The results of this study have important policy implications with respect to publicly funded health expenditures for the Indian states. The Indian states could rapidly achieve better health goals by spending more on their health sector. Given the health needs of Indian states, the study advocates an enhancement of publicly financed health expenditure and improving health infrastructure among the Indian states. Based on future data availability, this study can be extended to examine the contribution of human resources such as social workers, community workers, and paramedical staff for achieving specific health care goals related to child health, maternal health, and other types of physical and mental health in India.

## Supplementary Information

**Additional file 1. Table S1**: Definition and data source of variables. **Table S2**: Results of panel unit root tests.

## Data Availability

Data will be available upon request.

## Abbreviations

NHM: National Health Mission
SDG: Sustainable Development Goals
PHE: Publicly financed expenditure on health
NRHM: National Rural Health Mission
RSBY: Rashtriya Swasthya Bima Yojana
UHC: Universal Health Coverage
NUHM: National Urban Health Mission
PCPHE: Per capita publicly financed health expenditure
GSDP: Gross State Domestic Product
IMR: Infant Mortality Rate
CMR: Child Mortality Rate
HFS: High-Focus States
NHFS: Non-High Focus States
GSDP: Gross State Domestic Product
LE: Life Expectancy at birth
RBI: Reserve Bank of India
CSO: Central Statistics Office
EPWRF: EPW Research Foundation
SRS: Sample Registration System
ORGCC: Office of the Registrar General & Census Commissioner
GOI: Government of India
PCGSDP: Per capita GSDP (PCGSDP)
RE: Random-effects
FE: Fixed-effects
GCS: General Category States
SCS: Special Category States
SC: Sub-Centres
PHC: Primary Health Centre
CHC: Community Health Centre

## Footnotes

1 The MDGs were eight international development goals for the year 2015 that had been established following the Millennium Summit of the United Nations in 2000, following the adoption of the United Nations Millennium Declaration.

2 The main aim of this mission was to complement/support health expenditure in states, particularly poor-performing states. Thus, the Central Government plays a supportive role to States to provide equitable, affordable and quality health care services in each state.

3 The recently constituted National Health Policy (2017) also recommended an increase in health expenditure by the government as a percentage of GDP from the existing 1.15 per cent to 2.5 per cent by 2025. Various five-year plans like the 10th, 11th, and 12th have also taken steps to enhance publicly financed health expenditure in India.

4 NRHM and NUHM are subsumed under one broad central sponsored scheme named National Health Mission (NHM) in India.

5 It will cover ten crore poor and vulnerable families by providing insurance coverage up to five lakh rupees per family in a year for secondary and tertiary care hospitalization.

6 India has been lagging on targets to reduce child and infant mortality (Goal 4); improve maternal health (Goal 5); improving access to adequate sanitation facilities (goal 7). *Source*: https://www.unescap.org/sites/default/files/India_and_the_MDGs_0.pdf.

7 Health is in the concurrent list of the Indian constitution, which is the joint domain of the Central and State Governments.

8 Central Government accounts for nearly one-third of India's publicly financed health expenditure.

9 This study has focused only on publicly financed health expenditure because time series data on private health expenditure across the Indian states are unavailable, and publicly funded health expenditure is an important policy variable.

10 Telangana state is excluded from the analysis as it was founded in June 2014. In India, the financial year begins in April and ends in March. Therefore, the year 2000 means 2000–2001 and so on.

11 The NHM is a centrally sponsored scheme comprising two sub-mission, i.e., National Rural Health Mission launched in April 2005, and the National Urban Health Mission introduced in May 2013. The primary objective is to provide accessible, affordable, and quality health care to all in the country.

12 The High-Focus States (HFS) are Bihar, Chhattisgarh, Himachal Pradesh, Jammu and Kashmir, Jharkhand, Madhya Pradesh, Odisha, Rajasthan, Uttar Pradesh, Uttarakhand, Arunachal Pradesh, Assam, Manipur, Meghalaya, Mizoram, Nagaland, Sikkim, and Tripura. The Non-high Focus States (NHFS) include Andhra Pradesh, Goa, Gujarat, Haryana, Karnataka, Kerala, Maharashtra, Punjab, Tamil Nadu, and West Bengal. HFS has relatively poor health indicators and also poor infrastructure than the NHFS. NHM funds were primarily meant to support health expenditure in poor-performing states (i.e., HFS). Thus, a separate analysis for these two groups is being carried out to verify the objective.

13 It includes Medical and Public health and Family Welfare expenditure from current and capital accounts of the respective State budget.

14 It is derived by adding the total number of Sub-Centres (SC), Primary Health Centre (PHC), and Community Health Centre (CHC). Then further it is divided by per 1000 population.

15 IMR is the number of infants dying under one year per 1000 live births.

16 It is the probability per 1000 live births that a newborn baby will die before reaching age five. Here, 0–4 years of age (mortality) is considered.

17 The total number of malaria cases reported during a year. The incidence of malaria is calculated as per 1000 population.

18 It is the average of four major types of immunization, i.e., BCG immunization for children achieved, Measles immunization achieved, Polio immunization achieved, and Tetanus immunization for expectant mothers achieved. Additionally, we have done separate estimations for individual immunization programs. All variables are in the per capita population term and value is represented in rate (per 1000 population).

19 Including other factors that affect the health outcome might yield a more precise estimate of the relationship between publicly financed health care expenditure and outcome.

20 Due to data constraints, we cannot add other crucial variables like mother’s education/literacy, private health expenditure, culture, inequality, governance, etc., in the estimated models. Except for our variable of interest, we have taken per capita income (a strong relationship with other factors such as education, poverty, inequality, nutrition, etc.) and health infrastructure as a proxy for other leftover variables influencing health outcomes.

21 We have done the empirical analysis using the fixed effects/random effects regression model based on the Hausman test. This statistical method helps in controlling for time-invariant unobservable characteristics of each state. These characteristics of states are very difficult to measure; if we ignore them, the regression might face the problem of omitted variable bias.

22 We converted the variables into logarithmic transformation due to the following reasons. First, the log–log specification standardizes the data by reducing skewness because regression can be influenced significantly by outliers of one or both variables. Given the nature of variables used in the selected panel regressions, we prefer to use the log form. Second, it helps in measuring the elasticity and makes it easy to interpret the coefficient values as percentage changes of one variable with respect to one or more variables.

23 These are the most widely accepted health indicators by demographers, health economists, and policymakers.

24 However, the coefficients of other models are almost similar, which shows that the empirical estimations are robust.
