# Supplementary material for: Heterogeneity in health funding and disparities in health outcome: a comparison between high focus and non-high focus states in India
Source: Cost Eff Resour Alloc. 2023 Jul 17;21:44. doi: 10.1186/s12962-023-00451-x (PMC10351161; doi:10.1186/s12962-023-00451-x)
Supplement: Supplementary file 1 — Additional file 1. Table S1: Definition and data source of variables. Table S2: Results of panel unit root tests. [file 12962_2023_451_MOESM1_ESM.docx]

**Additional File 1**

**Table S1. Definition and Data Source of Variables**

| Variables | Definition |
| --- | --- |
| LE | Life Expectancy at Birth |
| IMR | Infant Mortality Rate |
| CMR | Child Mortality Rate |
| MALARIA | Incidence of malaria (per 1000 population) |
| IMMU | Per capita Immunization coverage rate |
| BCG | Per capita BCG vaccine achieved rate |
| MEASLES | Per capita Measles vaccine achieved rate |
| POLIO | Per capita Polio vaccine achieved rate |
| TETANUS | Per capita Tetanus vaccine for expectant mothers achieved rate |
| PCPHE | Per capita publicly financed health expenditure |
| PCGSDP | Per capita Gross State Domestic Product |
| INFRA | Total health services infrastructure (per 1000 population) |

Note: Immunization coverage includes an average of BCG, Polio, Measles, and Tetanus; Rate means per 1000 population.

Source: Author’s calculations.

**Table S2. Results of Panel Unit Root Tests**

| Variables | Levin, Lin & Chu (LLC) | | Im, Pesaran, and Shin (IPS) | |
| --- | --- | --- | --- | --- |
|  | (level) | (1^st^Diff.) | (level) | (1^st^Diff.) |
| lnLE_it_ | 3.993*** | -10.593*** | -0.057 | -3.602*** |
| lnIMR_it_ | -7.424*** | -7.942*** | -2.021** | -1.829** |
| lnCMR_it_ | -8.150*** | -14.161*** | -3.532*** | -5.455*** |
| lnMALARIA_it_ | -7.773*** | -11.614*** | -2.684*** | -3.748*** |
| lnIMMU_it_ | -9.002*** | -16.285*** | -2.265** | -5.996*** |
| lnPCPHE_it_ | -4.234*** | -15.260*** | 0.174 | -5.814*** |
| lnPCGSDP_it_ | -7.595*** | -13.438*** | -2.475** | -5.265*** |
| lnINFRA_it_ | -5.155*** | -14.897*** | 0.050 | -4.993*** |
| lnBCGI_it_ | -7.478*** | -14.664*** | -1.414* | -5.504*** |
| lnPOLIO_it_ | -10.379*** | -20.609*** | -3.332*** | -6.588*** |
| lnTETANUS_it_ | -14.348*** | -18.624*** | 5.844*** | -7.123*** |
| lnMEASLES_it_ | -7.386*** | -15.252*** | -2.264** | -6.302*** |

Note: ln: Natural logarithm; *** p<0.01, ** p<0.05, * p<0.1.

Source: Author’s calculations.
